# Novel Orthonairovirus Isolated from Ticks near China–North Korea Border

**DOI:** 10.3201/eid2906.230056

**Published:** 2023-06

**Authors:** Fan Li, Jixu Li, Jingdong Song, Qikai Yin, Kai Nie, Songtao Xu, Ying He, Shihong Fu, Guodong Liang, Qiang Wei, Huanyu Wang

**Affiliations:** Chinese Center for Disease Control and Prevention, Beijing, China (F. Li, J. Song, Q. Yin, K. Nie, S. Xu, Y. He, S. Fu, G. Liang, Q. Wei, H. Wang);; Yanbian Korean Autonomous Prefecture Center for Disease Control and Prevention, Jilin, China (J. Li)

**Keywords:** Orthonairovirus, viruses, genetic identification, morphology, China–North Korea border, ticks, vector-borne infections, China

## Abstract

We isolated a new orthonairovirus from *Dermacentor silvarum* ticks near the China–North Korea border. Phylogenetic analysis showed 71.9%–73.0% nucleic acid identity to the recently discovered Songling orthonairovirus, which causes febrile illness in humans. We recommend enhanced surveillance for infection by this new virus among humans and livestock.

Viruses of the genus *Orthonairovirus*, family *Nairoviridae*, include the consequential tick-transmitted pathogens Crimean–Congo hemorrhagic fever virus and Nairobi sheep disease virus, as well as other poorly characterized viruses that have been found in ticks and mammals. *Orthonairovirus* virions are spherical in shape (80–120-nm diameter) with 3 single-stranded RNA segments 17.1–22.8 kilobases in length and a membrane envelope (*1*–*5*). We performed surveillance in areas endemic for tick-borne encephalitis (*6*) and identified a novel orthonairovirus from *Dermacentor silvarum* ticks collected in 2021 in Jilin Province, China, near the China–North Korea border. 

## The Study 

On April 17, 2021, we dragged corduroy to collect ticks from a forest region in Antu (118°46′E, 43°15′N), a district of the city of Yanbian in eastern Jilin Province, China, near the border with North Korea. We identified captured ticks according to morphologic keys and stored them at 4°C with wet cotton. We collected 264 ticks of 3 species—29 *Ixodes persulcatus*, 193 *Dermacentor silvarum*, and 12 *Haemaphysalis concinna*—and 30 larvae of unidentified species. 

**Figure 1:**
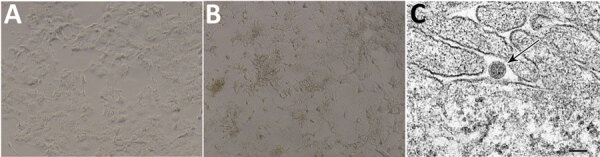
Discovery and characterization of novel orthonairovirus Antu virus isolate YB_tick_2021_24 from *Dermacentor silvarum* ticks in China. A) Vero E6 cells without YB_tick_2021_24 infection. Original magnification ×10. B) YB_tick_2021_24-infected Vero E6 cells showing cytopathic effects visible by light microscopy. Original magnification ×10. C) Ultrathin section electron micrograph of an isolated particle (black arrow) on a cell surface. Scale bar = 100 nM

We homogenized ticks using a QIAGEN TissueLyser (QIAGEN, https://www.qiagen.com) and inoculated supernatants onto a monolayer of African green monkey kidney (Vero) E6 cells. After 3 successive passages, we observed cells for cytopathic effects. The inoculate from *Dermacentor silvarum* ticks, designated as YB_tick_2021_24, caused cytopathic effects in Vero E6 cells 96 h after inoculation (Figure 1, panels A, B). We collected cells showing cytopathic effects, then fixed and embedded them in epoxy resin. We cut ultrathin (80 nm) sections from the resin block, stained them with citrate lead and uranyl acetate, and observed them under a transmission electron microscope. We observed enveloped virus particles ≈100 nm in diameter that shared morphologic features with *Bunyavirales* viruses (Figure 1, panel C).

We extracted viral RNA from infected culture supernatants using a QIAGEN QIAamp Viral RNA Mini Kit, synthesized cDNA, prepared DNA libraries using an Illumina Nextera XT Kit (Illumina, https://www.illumina.com), and performed 150 bp paired-end sequencing using the Illumina MiniSeq System. We filtered reads on the basis of their length and mean quality values. We prepared contigs by de novo assembly and subjected them to BLASTx alignment (https://blast.ncbi.nlm.nih.gov/Blast.cgi) at E value <10^−4^ against the nonredundant protein and viral proteome databases of the National Center for Biotechnology Information. We used Bowtie 2 (https://bowtie-bio.sourceforge.net/bowtie2/index.shtml) to remap the clean reads to the generated virus-related contigs (*7*). We used rapid amplification of cDNA ends (RACE) PCR and Sanger sequencing to confirm the terminal sequences of virus genomes, and deposited the new genome in GenBank (accession nos. OQ207701–3). We identified open read frames (ORFs) using ORF finder (https://www.ncbi.nlm.nih.gov/orffinder) and calculated sequence similarities using BLAST. 

Our procedure generated 40,826,350 reads (6.1 Gbp), which produced 266 virus-related contigs. Three contigs, the 1,516 bp small (S), 3,936 bp medium (M), and 12,133 bp large (L) segments, were annotated to Songling virus (SLV), a previously reported orthonairovirus (*8*). Average sequencing coverages remapped to the 3 contigs were 48× (S), 63× (M), and 234× (L). The final genome lengths confirmed by RACE sequencing were 1,848 bp encoding 488 aa for the S segment, 4,099 bp encoding 1,263 aa for the M segment, and 12,001 bp encoding 3,950 aa for the L segment. We performed multiple alignments using MAFFT version 7 (https://mafft.cbrc.jp/alignment/server) (*9*) and constructed a phylogenetic tree in MEGA7 (https://www.megasoftware.net) by using the neighbor-joining method with a bootstrap test for 1,000 replicates (*10*).

**Figure 2:**
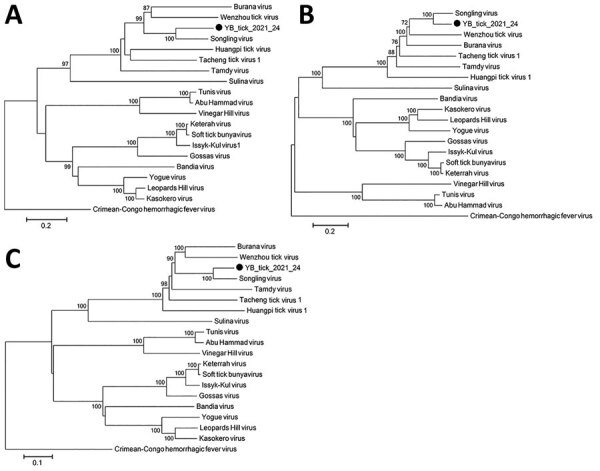
Molecular phylogenetic analysis by neighbor-joining tree based on the amino acid sequences novel orthonairovirus Antu virus isolate YB_tick_2021_24 (black circles) from *Dermacentor silvarum* ticks in China. A) Small segment; B) medium segment; C) large segment. Numbers associated with branches indicate percentages of 1,000 bootstrap replicates that support the existence of these branches. Branches with <70% bootstrap support have been collapsed. Scale bars represent amino acid substitutions per site.

Phylogenetic analysis showed the strain belongs to the genus *Orthonairovirus*, family *Nairoviridae*, and is genetically related to SLV (Figure 2) (*4**,**5**,**8**,**11*). The terminal nucleotides of the S segment were identical to those of orthonairoviruses (3′ segment terminus AGAGUUUCU and 5′ segment terminus AGAAACUCU) (*5*). The termini of the M and L segments were different (Appendix Figure). Homology analysis comparing YB_tick_2021_24 with SLV sample YC585 showed 71.9% nucleic acid (na) and 71.5% aa identities for the S segment, 72.4% na and 79.5% aa identities for the M segment, and 73.0% na and 84.6% aa identities for the L segment (Table) (*8*). Those results indicate that the isolate represents a unique *Orthonairovirus* species. For purposes of archiving, we designated novel YB_tick_2021_24 as Antu virus and deposited the strain in the National Pathogen Resource Center (accession no. NPRC 2.3.9401). 

**Table:** Homology comparisons of the sequence of novel orthonairovirus Antu viruses from China and other related viruses*

Protein/virus	Antu virus	SLGV	WTV	TTV	BURV	TDYV	HTV	CCHFV
Small								
Antu virus		71.5	52.6	53.7	52.1	48.9	47.4	37.3
SLGV	71.9		55.3	51.2	51.8	49.6	45.9	34.9
WTV	60.3	60.6		50.0	54.1	43.8	46.0	34.0
TTV	59.5	57.9	56.6		46.6	49.1	51.0	33.4
BURV	58.6	59.9	60.5	55.2		44.3	42.4	35.0
TDYV	56.5	58.2	54.0	57.9	53.1		45.0	34.3
HTV	55.5	55.8	55.9	58.7	52.9	55.8		35.2
CCHFV	47.6	46.8	46.8	46.5	47.9	47.7	46.3	
Medium								
Antu virus		79.5	59.9	56.3	58.7	53.8	46.9	25.4
SLGV	72.4		58.2	53.9	57.0	51.2	46.5	24.5
WTV	61.9	61.8		51.8	54.0	50.0	46.5	24.5
TTV	57.2	58	56.4		51.6	51.1	48.1	24.5
BURV	61	61.2	58.9	58		49.4	47.2	24.0
TDYV	56.6	57.5	55.9	55.1	55.4		45.7	24.4
HTV	53.7	53.9	53.4	52.9	54.2	52.1		24.6
CCHFV	41.8	40.7	40.5	40.6	41.3	42.2	40.3	
Large								
Antu virus		84.6	66.5	64.1	66.0	62.2	60.1	39.2
SLGV	73.0		65.7	64.0	65.1	61.4	60.1	38.5
WTV	63.4	63.7		63.5	69.7	61.7	60.0	38.5
TTV	61.9	62.0	62.2		63.2	59.3	60.0	38.7
BURV	63.4	63.3	66.1	61.8		61.5	60.3	38.7
TDYV	60.4	60.5	60.5	59.4	60.8		58.0	39.2
HTV	59.8	60.1	59.5	61.5	60.3	58.2		38.2
CCHFV	48.1	48.3	48.2	48.0	48.8	48.1	48.5	

*Percentage nucleotide sequence identity presented below and amino acid identity above blank cells. BURV, Burana virus; CCHFV, Crimean-Congo hemorrhagic fever virus; HTV, Huangpi tick virus; SGLV, Songling virus; TDYV, Tamdy virus; TTV, Tacheng tick virus; WTV, Wenzhou tick virus.

## Conclusion

We identified a novel orthonairovirus, Antu virus, in *Dermacentor silvarum* ticks collected in China near the China–North Korea border. Nucleotide and amino acid sequence homologies, combined with phylogenetic analysis of other orthonairovirus genomes, suggested that Antu virus is a new member of the genus *Orthonairovirus*, genetically related to SLV. Tamdy virus and SLV are orthonairoviruses reportedly able to infect human and livestock (*8*,*12*,*13*). Lacking direct evidence of the ability of Antu virus to infect and cause illness among humans and livestock animals, we recommend enhanced monitoring and surveillance for Antu virus infection among humans and livestock in potentially endemic areas. 

Appendix Additional information on a new orthonairovirus species isolated from *Dermacentor silvarum* ticks near the China–North Korea border.

## References

[1] Garrison AR, Alkhovsky Альховский Сергей Владимирович SV, Avšič-Županc T, Bente DA, Bergeron É, Burt F, et al. ICTV virus taxonomy profile: *Nairoviridae.* J Gen Virol. 2020;101:798–9. 10.1099/jgv.0.001485 32840475 PMC7641396

[2] Lasecka L, Baron MD. The molecular biology of nairoviruses, an emerging group of tick-borne arboviruses. Arch Virol. 2014;159:1249–65. 10.1007/s00705-013-1940-z 24327094 PMC7087186

[3] Bergeron É, Zivcec M, Chakrabarti AK, Nichol ST, Albariño CG, Spiropoulou CF. Recovery of recombinant Crimean Congo hemorrhagic fever virus reveals a function for non-structural glycoproteins cleavage by furin. PLoS Pathog. 2015;11:e1004879. 10.1371/journal.ppat.1004879 25933376 PMC4416775

[4] Walker PJ, Widen SG, Wood TG, Guzman H, Tesh RB, Vasilakis N. A global genomic characterization of nairoviruses identifies nine discrete genogroups with distinctive structural characteristics and host-vector associations. Am J Trop Med Hyg. 2016;94:1107–22. 10.4269/ajtmh.15-0917 26903607 PMC4856612

[5] Kuhn JH, Wiley MR, Rodriguez SE, Bào Y, Prieto K, Travassos da Rosa AP, et al. Genomic characterization of the genus *Nairovirus* (family *Bunyaviridae*). Viruses. 2016;8:164. 10.3390/v8060164 27294949 PMC4926184

[6] Chen X, Li F, Yin Q, Liu W, Fu S, He Y, et al. Epidemiology of tick-borne encephalitis in China, 2007- 2018. PLoS One. 2019;14:e0226712. 10.1371/journal.pone.0226712 31877145 PMC6932775

[7] Langmead B, Salzberg SL. Fast gapped-read alignment with Bowtie 2. Nat Methods. 2012;9:357–9. 10.1038/nmeth.1923 22388286 PMC3322381

[8] Ma J, Lv XL, Zhang X, Han SZ, Wang ZD, Li L, et al. Identification of a new orthonairovirus associated with human febrile illness in China. [Erratum in Nat Med. 2021;27:926.]. Nat Med. 2021;27:434–9. 10.1038/s41591-020-01228-y 33603240

[9] Katoh K, Standley DM. MAFFT multiple sequence alignment software version 7: improvements in performance and usability. Mol Biol Evol. 2013;30:772–80. 10.1093/molbev/mst010 23329690 PMC3603318

[10] Kumar S, Stecher G, Tamura K. MEGA7: Molecular Evolutionary Genetics Analysis version 7.0 for bigger datasets. Mol Biol Evol. 2016;33:1870–4. 10.1093/molbev/msw054 27004904 PMC8210823

[11] Zhou H, Ma Z, Hu T, Bi Y, Mamuti A, Yu R, et al. Tamdy virus in *Ixodid* ticks infesting bactrian camels, Xinjiang, China, 2018. Emerg Infect Dis. 2019;25:2136–8. 10.3201/eid2511.190512 31625865 PMC6810205

[12] Liu X, Zhang X, Wang Z, Dong Z, Xie S, Jiang M, et al. A tentative Tamdy orthonairovirus related to febrile illness in northwestern China. Clin Infect Dis. 2020;70:2155–60. 10.1093/cid/ciz602 31260510

[13] Moming A, Shen S, Fang Y, Zhang J, Zhang Y, Tang S, et al. Evidence of human exposure to Tamdy virus, northwest China. Emerg Infect Dis. 2021;27:3166–70. 10.3201/eid2712.203532 34808086 PMC8632163

