# Supplementary material for: Novel Orthonairovirus Isolated from Ticks near China–North Korea Border
Source: Emerg Infect Dis. 2023 Jun;29(6):1254–7. doi: 10.3201/eid2906.230056 (PMC10202851; doi:10.3201/eid2906.230056)
Supplement: Appendix — Additional information on a new orthonairovirus species isolated from Dermacentor silvarum ticks near the China–North Korea border. [file 23-0056-Techapp-s1.pdf]

Article DOI: https://doi.org/10.3201/eid2906.230056

EID cannot ensure accessibility for supplementary materials supplied by authors. Readers who have difficulty accessing supplementary content should contact the authors for assistance.

Novel Orthonairovirus Isolated from Ticks near China–North Korea Border

Appendix

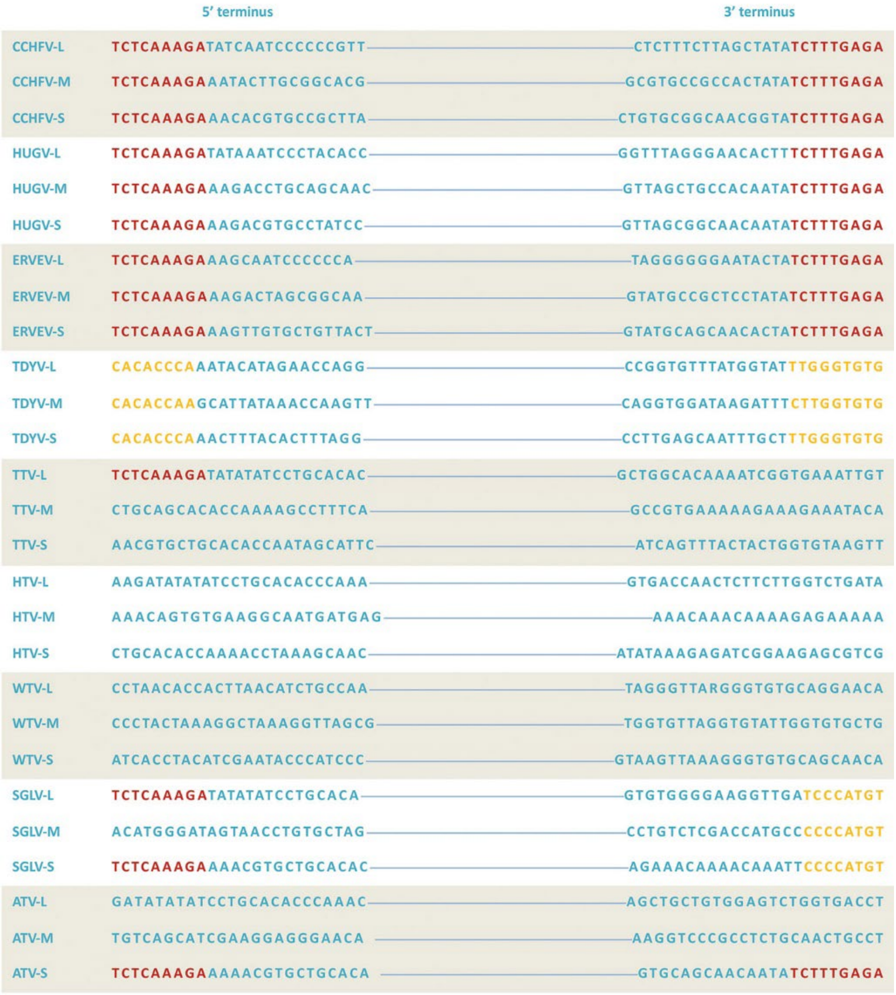

Appendix Figure. Terminal sequences of ATV and other orthonairoviruses. Typical terminal sequences of orthonairoviruses are highlighted in yellow and putative terminal sequences in red. L, Large segment;

M, medium segment; S, small segment; CCHFV, Crimean-Congo hemorrhagic fever virus (L, NC_005301; M, NC_005300; S, NC_005302); HUGV, Hughes virus (L, NC_040512; M, NC_040513; S, NC_040514); ERVEV, Erve virus (L, JF911697; M, JF911698; S, JF911699); TDYV, Tamdy virus (L, MK757580; M, MK757581; S, MK757582); TTV, Tacheng tick virus 1 (L, NC_031284; M, NC_031285; S, NC_031286); HTV, Huangpi tick virus 1 (L, NC_031135; M, NC_031136; S, NC_031137); WTV, Wenzhou tick virus (L, NC_031291; M, NC_031288; S, NC_031289); SGLV, Songling virus (L, MT328776; M, MT328775; S, MT328777); ATV, Antu virus.
